# Phaseless Terahertz Coded-Aperture Imaging for Sparse Target Based on Phase Retrieval Algorithm

**DOI:** 10.3390/s19214617

**Published:** 2019-10-23

**Authors:** Long Peng, Chenggao Luo, Bin Deng, Hongqiang Wang, Shuo Chen, Jun Dong

**Affiliations:** 1 College of Electronic Science and Technology, National University of Defense Technology, Changsha 410073, China; penglong_nudt@163.com (L.P.); dengbin_nudt@163.com (B.D.); oliverwhq@tom.com (H.W.); chenshuo_nudt@163.com (S.C.); 2 College of Information Science and Engineering, Hunan Normal University, Changsha 410081, China; jdongcn@outlook.com

**Keywords:** terahertz, coded-aperture imaging, phaseless imaging, phase retrieval, Wirtinger flow, optimal stepsize

## Abstract

Phaseless terahertz coded-aperture imaging (PL-TCAI) is a novel radar computational imaging method that utilizes the coded aperture and the incoherent detector array to achieve forward-looking and high-resolution imaging without relying on relative motion. In this paper, we propose a more reasonable and compact architecture for the PL-TCAI system and derive the imaging model of PL-TCAI based on the random frequency-hopping signal. Since most phase retrieval algorithms for PL-TCAI utilize only the intensity of echo signals to accurately reconstruct the target, excessive measurement samples are usually required. In order to reduce the number of measurement samples required for imaging, this paper proposes a sparse Wirtinger flow algorithm with optimal stepsize (SWFOS) by using the sparse prior of the target. The specific procedures of the SWFOS algorithm include the support recovery, initialization by truncated spectral method, iteration via gradient descent scheme, hard threshold operation, and stepsize optimization of iteration. Numerical simulations are performed, and the results show that the SWFOS algorithm not only has good performance for the PR problem, but can also sharply reduce the number of measurement samples required for imaging in the PL-TCAI system.

## 1. Introduction

Terahertz coded-aperture imaging (TCAI) [1,2,3,4] is a novel high-resolution imaging method that does not rely on any relative motion between the radar and the target. In TCAI, the computer-controlled electronic coded aperture [5,6,7,8] can modulate the amplitude or phase of the transmitting signal to form a space–time two-dimensional random orthogonal radiation field, thereby randomly generating various illumination modes in the imaging area. Random radiation fields can help to improve radar imaging resolution, even though they are accompanied by a significant reduction in detection distance [9]. The azimuth resolution of TCAI is not related to the Doppler frequency, so it does not depend on the relative motion with the target, which can achieve forward-looking and staring imaging [9,10]. For the TCAI system, the reference signal corresponding to the imaging area can be derived and then combined with the receiving signal, and then the matrix imaging equation of TCAI can be established. In recent years, with the increasing demand for forward imaging and high-resolution imaging, TCAI has received more and more attention. For example, since the frequency range of terahertz waves is 0.1–10 THz, TCAI can be used for human security screening at airports, stations, or other important places, and it does no harm to the human body compared to the X-ray screening machines [11,12,13,14]; TCAI can also be used as a forward-looking sensor for autonomous vehicles [15].

For traditional TCAI with coherent receivers, high frequency and wide bandwidth signals are beneficial for improving imaging resolution. However, in the coherent detection of terahertz signals with high frequency and wide bandwidth, the receiver still faces many challenges [16,17], where the down-conversion circuit includes a low noise amplifier, a local oscillator, and a mixer [18]. Coherent receivers have complex circuits and high costs, and especially when it is desired to speed up reception by multiple-output technology, expensive coherent receivers can multiply the complexity and cost of the TCAI system. Aside from that, accurate phase measurement of the high-frequency signal is still difficult at present, and the influence of phase error on imaging is greater than the intensity error [19].

Accordingly, in this paper, we propose a more compact PL-TCAI structure, which adopts a combination of an incoherent detector array and an electronic-coded aperture. The proposed PL-TCAI replaces the coherent receiver of traditional TCAI with an incoherent detector array, eliminating the need for the down-conversion circuits or bandwidth reduction components, which makes the system simpler and more cost-effective. In addition, the incoherent detector array, which utilizes multiple-output technology, can accelerate the imaging speed and reduce the number of coding and sampling compared to a single detector; therefore, it is capable of moving-target imaging or real-time imaging. PL-TCAI only detects the intensity of the echo signal to establish the phaseless imaging equation. Solving the imaging equation can be regarded as a phase retrieval (PR) problem, and in Reference [20], we have proved that the PR algorithm can be adopted to accurately reconstruct the target in PL-TCAI. 

PR algorithms can reconstruct signals from phaseless measurements, and they have wide applications in many fields, such as optics, X-ray crystallography, astronomical imaging, and radar imaging [21,22,23]. As classical PR algorithms, the Gerchberg–Saxton (GS) algorithm [24] and its variants correct estimates by alternating projections between time and frequency domains with some special constraints, but they often fall into local minimums. Semidefinite programming (SDP) based algorithms, such as the PhaseLift algorithm [25] based on matrix lifting, are convex optimization methods. However, due to matrix lifting, the computational complexity of the PhaseLift algorithm will increase sharply as the dimension of the signal increases. The Wirtinger Flow (WF) [26] algorithm is a nonconvex algorithm, which has attracted more and more attention in recent years. It mainly adopts the gradient descent method to iteratively update estimates and finds the global minimum. In fact, the WF algorithm is sensitive to the number of samples and requires too many samples to solve the problem accurately. Based on the WF algorithm, variant algorithms such as the Truncated Wirtinger Flow (TWF) [27] and Incremental Truncated Wirtinger Flow (ITWF) [28] algorithms are designed to reduce the amount of sample required for the calculation. In addition, nonconvex algorithms based on amplitude cost function, such as the Reshaped Wirtinger Flow (RWF) [29], Truncated Amplitude Flow (TAF) [30], and Reweighted Amplitude Flow (RAF) [31] algorithms, are more significant in reducing the amount of sample because they have non-smooth but simpler loss functions. Further research also includes sparse PR algorithms, such as the Thresholding Wirtinger Flow [32], Sparse Phase Retrieval via Truncated Amplitude (SPARTA) [33] and Sparse Wirtinger Flow (SWF) [34] algorithms, which can accurately recover solutions with a small number of samples utilizing sparse prior.

In this paper, based on the SPARTA, SWF, Wirtinger Flow with Optimal Stepsize (WFOS) [35], and Thresholding Wirtinger Flow algorithms, we propose a sparse Wirtinger flow algorithm with optimal stepsize (SWFOS) for PL-TCAI. The proposed SWFOS algorithm can utilize the sparse priori of the target to effectively reduce the number of measurement samples required in PL-TCAI, significantly reduce the number of coding and sampling, and further improve the imaging speed. The specific procedures of the SWFOS algorithm include the support recovery, initialization by truncated spectral method, iteration via gradient descent scheme, hard threshold operation, and stepsize optimization of iteration. The stepsize selection is especially critical to the PR algorithms based on the gradient descent method, which directly affects the performance of the algorithms. If the stepsize is too large, the algorithms will diverge easily, while if the stepsize is too small, the algorithms will converge slowly. For the SPARTA, SWF, and Thresholding Wirtinger Flow algorithms, the stepsizes are selected empirically, and the default stepsize functions or stepsize constants are proposed for the measurement vectors that conform to the standard Gaussian random distribution [26,27,28,29,30,31,32,33,34,35]. However, the measurement vectors of PL-TCAI conform to sub-Gaussian random distribution, and the stepsize selection criteria or functions based on the standard Gaussian random distribution are no longer applicable to PL-TCAI. Different PL-TCAI systems may require different stepsize selection criteria, and it is undoubtedly difficult to choose a new stepsize for PL-TCAI based on experience or repeated experiments, which is almost impossible to achieve in the practical PL-TCAI systems. Therefore, the optimal stepsize operation is necessary in the different PL-TCAI systems, which avoids the empirical selection of stepsize by calculating the stepsize of each iteration.

This paper is organized as follows. In Section 2, a new PL-TCAI configuration, which combines the incoherent detector array with the electronic coded aperture, is introduced in detail, and the imaging model of PL-TCAI based on the random frequency-hopping (R-FH) signal is derived. Section 3 analyzes the target reconstruction principle of PL-TCAI and proposes a sparse PR algorithm for PL-TCAI, in which the specific procedure of the SWFOS algorithm is given. In Section 4, numerical simulations are implemented to demonstrate the general performance of the SWFOS algorithm in the PR problem and the feasibility of the SWFOS algorithm to reconstruct targets in the proposed PL-TCAI. Finally, we summarize the main work and contributions of this paper in Section 5.

## 2. System Configuration and Imaging Model

### 2.1. Proposed PL-TCAI Architecture

**Figure 1:**
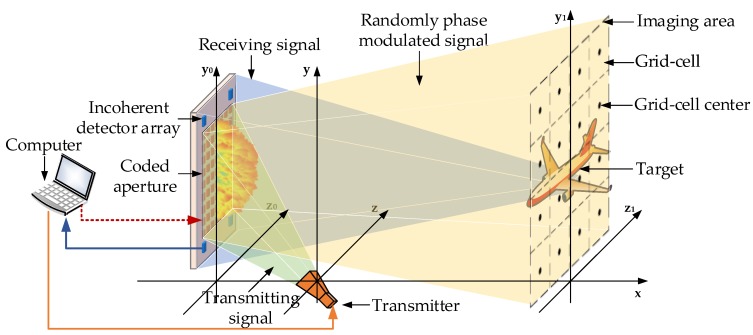
The architecture of the phaseless terahertz coded-aperture imaging (PL-TCAI) system based on combination of incoherent detector array and reflectarray coded aperture.

The configuration of our proposed PL-TCAI system is shown in Figure 1. It mainly includes a computer, a transmitter, a reflectarray coded aperture with an incoherent detector array. The computer is used to control different devices and to process echo data for imaging. During the transmitting process, the computer controls the transmitter to generate a specified form of terahertz signal and simultaneously loads the random modulation scheme to the coded aperture to modulate the terahertz wave. In the receiving process, the computer is used for computational imaging by processing the intensity data of the echo signal collected by the incoherent detector array. The transmitter is designed to emit terahertz continuous waves with a certain beamwidth of the main lobe, ensuring that it can sufficiently illuminate the entire coded aperture over a distance, while the side lobes need to be low enough to not affect the transmitted signal. Due to the different materials and modulation principles, the coded aperture usually has two modulation modes for terahertz waves to control their spatiotemporal independence, namely random phase modulation and random amplitude modulation. In this PL-TCAI system, a reflectarray coded aperture made of metamaterials [36,37,38] is selected for random phase modulation, which has proven to be practical in the terahertz band. The reflectarray coded aperture has the advantages of scalability, simple fabrication, and low cost [39]. It can achieve a wide range of phase shifts, and is also effective for large bandwidth signals. Here, we adopt a 1-bit digitally controlled reflectarray coded aperture [37], which is more convenient to load coding factors repeatedly and quickly. The reflectarray coded aperture is shown in the red block in Figure 1, which modulates terahertz waves into the spatiotemporal-independent radiation field in the imaging area. The incoherent detector array, which can only detect the intensity of the echo signal without phase information, is designed to be combined with the reflectarray coded aperture, as shown in the blue block in Figure 1. This design makes the PL-TCAI system more compact and integrated. Each incoherent detector element can independently transfer the intensity information of the echo signal to the computer for processing, which is inspired by multiple-output technology. The acquisition speed of the echo signal increases with the number of incoherent detector elements, but it should be noted that, in order to ensure the independence between different rows of the reference–signal matrix, the distance between the elements must be large enough. It is assumed that the target is located in an imaging area divided into *W* grid cells according to system resolution, and the strong scatterers of the target are exactly at the center of the grid cells. When the target appears in the imaging area, it changes the scattering coefficients corresponding to the grid cells. The purpose of the PL-TCAI system is to calculate the scattering coefficients of all grid cells in the imaging area so that the specific position, shape, and size of the target in the imaging area can be obtained from the distribution of the scattering coefficients. Obviously, the size and number of grid cells are the representation of imaging resolution and imaging area size, respectively.

### 2.2. Imaging Model

According to Figure 1, the imaging model of the proposed PL-TCAI can be deduced in detail. In the transmitting process, the signal transmitted by the transmitter may be a single-frequency signal, a step-frequency signal, or a random frequency-hopping (R-FH) signal. Here, assuming the PL-TCAI system adopts a terahertz R-FH signal, it can be written as Equation (1):

(1) $$s(t_{n})=A\exp\left[j2\pi \left(f_{c}+f_{t_{n}}\right)t_{n}\right],$$

where $s(t_{n})$ is the transmitting signal at time $t_{n}$, A is the amplitude of the signal, j is the imaginary unit, $f_{c}$ is the carrier frequency, and $f_{t_{n}}$ is the random frequency hopping at time $t_{n}$. The reflectarray coded aperture includes M phase modulation elements, and the position vector corresponding to the *m*-th element is $r_{m}$. The transmitting signal $s(t_{n},r_{m})$ arriving at the *m*-th coded aperture element can be expressed as Equation (2):

(2) $$s(t_{n},r_{m})=A\exp\left[j2\pi \left(f_{c}+f_{t_{n}}\right)\left(t_{n}-\frac{\left|r_{m}-R_{0}\right|}{c}\right)\right],$$

where $R_{0}$ is the position vector of transmitter, and c is the propagation speed of the wave. At the coded aperture, the transmitting signal is randomly phase modulated by the array elements. Controlled by the computer, the random phase modulation factor $\varphi_{m}\left(t_{n}\right)$ corresponding to the *m*-th element at time $t_{n}$ is loaded into the coded aperture. Then, the imaging area is divided into W grid cells, and the center position vector of the *w*-th grid cell is $r_{w}$. The incoherent detector array has a total of Q elements, and the *q*-th element position vector is $r_{q}$. Each detector element can detect the intensity of the signal and transmit it back to the computer for processing, according to the multiple-output technology. Hence, at time $t_{n}$, for the echo signal detected by the *q*-th detector array element, the reference signal $S^{\left(q\right)}(t_{n},r_{w})$ of the *w*-th imaging grid cell can be expressed as Equation (3):

(3) $$S^{\left(q\right)}(t_{n},r_{w})=\sum_{m=1}^{M}A\exp\left\{j\left[2\pi \left(f_{c}+f_{t_{n}}\right)\left(t_{n}-\frac{\left|r_{m}-R_{0}\right|}{c}-\frac{\left|r_{w}-r_{m}\right|}{c}-\frac{\left|r_{q}-r_{w}\right|}{c}\right)+\varphi_{m}\left(t_{n}\right)\right]\right\}.$$

Then, the intensity of the echo signal received by the *q*-th detector element at time $t_{n}$ can be expressed as Equation (4):

(4) $$Sr^{\left(q\right)}(t_{n})={\left|\sum_{w=1}^{W}\sum_{m=1}^{M}A\exp\left\{j\left[2\pi \left(f_{c}+f_{t_{n}}\right)\left(t_{n}-\frac{\left|r_{m}-R_{0}\right|}{c}-\frac{\left|r_{w}-r_{m}\right|}{c}-\frac{\left|r_{q}-r_{w}\right|}{c}\right)+\varphi_{m}\left(t_{n}\right)\right]\right\}\cdot \beta_{w}\right|}^{2},$$

where $\beta_{w}$ is the scattering coefficient corresponding to the *w*-th grid cell. According to Equation (6), we can derive the intensity of the echo signal received by the entire incoherent detector array at time $t_{n}$, which can be written as Equation (5):

(5) $$Sr(t_{n})={\left|S(t_{n})\cdot \beta \right|}^{2},$$

where $Sr(t_{n})={\left[Sr^{\left(1\right)}(t_{n}),Sr^{\left(2\right)}(t_{n}),\ldots ,Sr^{\left(Q\right)}(t_{n})\right]}^{T}$, and $\beta ={\left[\beta_{1},\beta_{2},\ldots ,\beta_{W}\right]}^{T}$ is the scattering coefficient vector. Here, ${\left[\cdot \right]}^{T}$ and $\left|\right.\cdot \left.\right|$ denote the matrix transposition and the absolute value, respectively. Aside from that, the reference signal matrix $S(t_{n})$ at time $t_{n}$ is written as Equation (6):

(6) $$S(t_{n})=\left[\begin{array}{cccc} S^{\left(1\right)}(t_{n},r_{1}) & S^{\left(1\right)}(t_{n},r_{2}) & \ldots & S^{\left(1\right)}(t_{n},r_{W})\\ S^{\left(2\right)}(t_{n},r_{1}) & S^{\left(2\right)}(t_{n},r_{2}) & \ldots & S^{\left(2\right)}(t_{n},r_{W})\\ \ldots & \ldots & \ldots & \ldots \\ S^{\left(Q\right)}(t_{n},r_{1}) & S^{\left(Q\right)}(t_{n},r_{2}) & \ldots & S^{\left(Q\right)}(t_{n},r_{W}) \end{array}\right].$$

After the frequency of the transmitting signal is randomly hopped *N* times and the coded aperture is loaded with the coding scheme *N* times, the intensity detector array samples the echo signal *N* times and obtains L=Q×N samples. Finally, in combination with Equation (5), the imaging model of PL-TCAI is deduced as

(7) $$Sr={\left|S\cdot \beta \right|}^{2}+\omega ,$$

where $Sr={\left[Sr(t_{1}),Sr(t_{2}),\ldots ,Sr(t_{N})\right]}^{T}$ and $S={\left[S(t_{1}),S(t_{2}),\ldots ,S(t_{N})\right]}^{T}$ are the final received signal vector and the final reference signal matrix, respectively. Furthermore, $\omega ={\left[\omega_{1},\omega_{2},\ldots ,\omega_{L}\right]}^{T}$ is the additive measurement noise vector at the receiving terminal.

As can be seen from Equations (6) and (7), for samples of the same size, sampling and coding with a single detector will be L=Q×N times, but when using a detector array with *Q* elements, there will be less sampling and coding, which will only take *N* times. Therefore, the proposed PL-TCAI with an intensity detector array can greatly reduce the number of sampling and coding, and directly improve the imaging efficiency.

Imaging processing is used to solve Equation (7) in order to accurately estimate β, which represents the target-scattering characteristics. Obviously, the imaging capability of PL-TCAI is affected by the structure of the reference signal matrix S. For PL-TCAI, an ideal S should be that all rows and columns are independent of each other. Fortunately, under the random phase modulation of the coded aperture, the columns of S can be independent of each other. In addition, by using the R-FH signal and increasing the distance between the detector elements, the independence of the rows of S can be increased.

## 3. Target Reconstruction Principle and SWFOS Algorithm

### 3.1. Target Reconstruction Principle

Reconstructing the target from Equation (7), where only the intensity of the echo signal is measured, is a typical PR problem [26,27,28,29,30,31,32,33,34,35]. Classic algorithms for solving PR problems include the GS algorithm, the PhaseLift algorithm, the WF algorithm, etc. The GS algorithm is highly dependent on a priori information about the target, and often gets stuck into local minimums without converging during the solution process. The PhaseLift algorithm solves a PR problem by establishing a higher-dimensional linear equation, which converts a non-convex problem into a convex problem. Although the PhaseLift algorithm can provide an accurate estimate, as the dimension of β increases, the memory required for the calculation will far exceed the capabilities of ordinary computers because of matrix lifting. However, for a non-convex problem of PR, the WF algorithm directly adopts the gradient descent method to iteratively update the estimated β, searching for the global minimum. In order to successfully and accurately estimate β, the WF algorithm often needs excessive samples, where the number of samples *L* may satisfy L/W≥6 [20,26]. 

As is well-known, obtaining a large number of samples is very unfriendly for a fast-imaging system. For the proposed PL-TCAI, although a detector array is adopted to speed up the detection of echoes, with the increase of the number of grid cells *W*, the number of samples *L* required by the WF algorithm becomes extremely large, thereby increasing the number of coding and sampling. In order to reduce the number of coding and sampling while increasing the speed of imaging, it is necessary to explore an algorithm that requires fewer samples. Recently, it was confirmed that utilizing the sparse prior information of the target can effectively reduce the samples required by the algorithm [33,34].

When the detected target is *k* sparse, it means that there are *k* strong scattering points in the imaging area. Therefore, for solving Equation (7), we can express the problem as the following:

(8) $$\mathrm{Find}\;\tilde{\beta }\;\mathrm{such}\;\mathrm{that}\;Sr_{i}={\left|S_{i}\tilde{\beta }\right|}^{2}+\omega_{i},\;{\left\|\tilde{\beta }\right\|}_{0}=k,$$

where $\tilde{\beta }$ is an estimated scattering coefficient vector, and $\left\|\right.\cdot {\left.\right\|}_{0}$ denotes the zero norm. $S_{i}$ is the vector of *i*-th row vector of S, and $Sr_{i}$ is the *i*-th element of Sr. Here, $\omega_{i}$ is the *i*-th measurement noise, and according to the central limit theorem (CLT) [40], the noise $\omega_{i}$ conforms to the Gaussian distribution, which can be written as $\omega_{i}∼N\left(0,\sigma \right)$. Therefore, the probability density function of $\omega_{i}$ can be written as the following:

(9) $$P\left(\omega_{i}\right)=\frac{1}{\sqrt{2\pi }\sigma }\exp\left(-\frac{{\left(\omega_{i}\right)}^{2}}{2\sigma^{2}}\right)=\frac{1}{\sqrt{2\pi }\sigma }\exp\left(-\frac{{\left(Sr_{i}-{\left|S_{i}\tilde{\beta }\right|}^{2}\right)}^{2}}{2\sigma^{2}}\right),i=1,2,\ldots ,L.$$

Here, the problem of solving the probability density function of $\omega_{i}$ in Equation (9) can be transformed into the problem of solving the estimated $\tilde{\beta }$, which can be solved by the following maximum likelihood function:

(10) $$\underset{\tilde{\beta }\in {\mathbb{R}}^{W}}{\mathrm{minimize}}f\left(\tilde{\beta }\right)=\frac{1}{2L}{\sum_{i=1}^{L}\left({\left|S_{i}\tilde{\beta }\right|}^{2}-Sr_{i}\right)}^{2},\;\mathrm{such}\;\mathrm{that}\;{\left\|\tilde{\beta }\right\|}_{0}=k.$$

In fact, Equation (10) is a non-convex quadratic system, which has many local minimums, and driving it to converge to a local minimum will be very difficult. This problem is customarily called non-deterministic polynomial hard. However, as mentioned in the previous section, the rows and columns of the reference signal matrix S obtained in PL-TCAI are independent of each other. In particular, each row of S is affected by the random phase modulation, resulting in a random distribution of the row vector $S_{i},i=1,2,\ldots ,L$, which has a benign geometrical structure. Hence, it is quite possible to find a global minimum from Equation (10) to obtain the estimated $\tilde{\beta }$.

### 3.2. SWFOS Algorithm

To search for the global minimum of Equation (10), we propose a sparse phase retrieval algorithm with the optimal stepsize (SWFOS) for fast iterative update. Recovering the support of β is arranged in the first step of the algorithm, and then we initialize the estimated ${\tilde{\beta }}_{0}$ via a truncated spectral method under the recovered supports. Finally, the estimated $\tilde{\beta }$ is iteratively updated using the gradient descent method, where each iteration adopts the optimal stepsize and adds a hard thresholding operation. The following is a detailed introduction to the SWFOS algorithm.

#### 3.2.1. Recovering the Support

It is assumed that the scattering coefficient vector conforms to $\beta \in {\mathbb{R}}^{W}$ and ${\left\|\beta \right\|}_{0}=k$, which represents that there are only *k* strong scattering points in the imaging area with *W* grid cells. Using the methods mentioned in References [33,34], we assume that β has a support $\beta^{\sup}$ that complies with $\beta^{\sup}\in {\mathbb{R}}^{W}$ and ${\left\|\beta^{\sup}\right\|}_{0}=k$. Here, the support $\beta^{\sup}$ is an index set, which is used to constrain the estimated $\tilde{\beta }$ during the initialization procedure. Besides, we define the random variables $Y_{i,j}=Sr_{i}S_{i,j}^{2}={\left|S_{i}\beta \right|}^{2}S_{i,j}^{2}$, where $S_{i,j}$ is the *j*-th element of $S_{i}$. Here, $S_{i}$ is the *i*-th row vector of the reference signal matrix S, and we assume that $S_{i}∼N\left(0,I_{W}\right)$. Thus, based on the moment of Gaussian variables, it can be calculated that $E\left[S_{i,j}^{4}\right]=3$ and $E\left[S_{i,j}^{2}\right]=1$. Finally, according to the rotational invariance property of Gaussian distributions, the following equation can be obtained:

(11) $$E\left[Y_{i,j}\right]=E\left[{\left|S_{i}\beta \right|}^{2}S_{i,j}^{2}\right]=E\left[S_{i,j}^{4}\beta_{j}^{2}+{\left(S_{/j}\beta_{/j}\right)}^{2}S_{i,j}^{2}\right]=3\beta_{j}^{2}+{\left\|\beta_{/j}\right\|}_{2}^{2},$$

where $\beta_{/j}$ is obtained by removing the *j*-th entry from β, while $S_{/j}$ is also obtained in the same way. Note that $E\left[Y_{i,j}\right]=2\beta_{j}^{2}+{\left\|\beta \right\|}_{2}^{2}$ will be valid at $j\in \beta^{\sup}$ and $\beta_{j}\neq 0$. Otherwise, if $j\notin \beta^{\sup}$ or $\beta_{j}=0$, there will be $E\left[Y_{i,j}\right]={\left\|\beta \right\|}_{2}^{2}$. With the help of the strong law of large numbers, we know that, when *L* is large enough, the sample average will approach the ensemble one. With the increase of *L*, it is easy to get the following:

(12) $${\tilde{Y}}_{i,j}:=\left(1/L\right)\sum_{i=1}^{L}Y_{i,j}=\frac{1}{L}\sum_{i=1}^{L}Sr_{i}S_{i,j}^{2}\to E\left[Y_{i,j}\right].$$

It can be seen from Equations (11) and (12) that ${\tilde{Y}}_{i,j}$ will be affected by $\beta_{j}$. For example, when $\beta_{j}^{2}$ is very large, the corresponding ${\tilde{Y}}_{i,j}$ will also be very large. It should be noted that $E\left[Y_{i,j}\right]=2\beta_{j}^{2}+{\left\|\beta \right\|}_{2}^{2}$ will be valid at $j\in \beta^{\sup}$ and $\beta_{j}\neq 0$. Therefore, we can sort out the largest *k* values from ${\left\{{\tilde{Y}}_{i,j}\right\}}_{j=1}^{W}$ and record their indices as the estimated support $\beta_{0}^{\sup}$. That is, the estimated support $\beta_{0}^{\sup}$ can be defined as $\beta_{0}^{\sup}=\left\{1\leq i\leq W\left|\mathrm{indices}\;\mathrm{of}\;\mathrm{the}\;\mathrm{largest}\;k\;\mathrm{instances}\;\mathrm{in}\;{\left\{{\tilde{Y}}_{i,j}\right\}}_{j=1}^{W}\right.\right\}$. In fact, it was proved in References [33,34] that if $S_{i}∼N\left(0,I_{W}\right),\;i=1,2,\ldots L$, the estimated support $\beta_{0}^{\sup}$ can be equal to the support $\beta^{\sup}$ with a probability of at least 1−6/L when $L\geq C_{0}k^{2}\log\left(LW\right)$ for some constant $C_{0}$ and minimum nonzero entries $\beta_{\min}:=\min_{j\in \beta^{\sup}}\left|\beta_{j}\right|$ on the order of $\left(1+\sqrt{k}\right)/{\left\|\beta \right\|}_{2}$. 

#### 3.2.2. Initialization via Truncated Spectral Method

Next, we need to initialize the estimated ${\tilde{\beta }}_{0}$ under the recovered support $\beta_{0}^{\sup}$. It can be noticed from Reference [26] that the spectral method is a very attractive initialization procedure, which initializes ${\tilde{\beta }}_{0}$ by calculating the leading eigenvector of $Y_{SM}=1/L\sum_{i=1}^{L}Sr_{i}\left(S_{i}{}^{T}S_{i}\right)$. If the initialized ${\tilde{\beta }}_{0}$ is sufficiently accurate, the estimated $\tilde{\beta }$ can be guaranteed to converge in the iterative update. However, this method is sensitive to the complexity of the sample, which needs to exceed WlogW. The smaller the sample complexity, the lower the success rate of initialization and the larger the relative error of initialization. Fortunately, in response to this shortcoming, an improved algorithm called the truncated spectral method is proposed in Reference [27], where the initialization is completed by calculating the leading eigenvector of $Y_{TSM}=1/L\sum_{i=1}^{L}Sr_{i}\left(S_{i}{}^{T}S_{i}\right)1_{\left\{\left|Sr_{i}\right|\leq \alpha_{y}^{2}\left(1/L\sum_{l=1}^{L}Sr_{l}\right)\right\}}$. Moreover, since the support $\beta_{0}^{\sup}$ of β is recovered in the previous section, we need to constrain $S_{i}$ by deleting some elements that are not in the support $\beta_{0}^{\sup}$. Thus, we can get a special matrix, which is written as the following:

(13) $$Y=\frac{1}{L}\sum_{i=1}^{L}Sr_{i}\left(S_{i,\beta_{0}^{\sup}}{}^{T}S_{i,\beta_{0}^{\sup}}\right)1_{\left\{\left|Sr_{i}\right|\leq \alpha_{y}^{2}\left(\frac{1}{L}\sum_{l=1}^{L}Sr_{l}\right)\right\}},$$

where $\alpha_{y}$ is a truncation threshold. Finally, after computing the leading eigenvector ${\tilde{\beta }}_{0,\beta_{0}^{\sup}}$ of Y, we need to set ${\tilde{\beta }}^{0}$ as $\sqrt{1/L\sum_{l=1}^{L}Sr_{l}}{\tilde{\beta }}_{0}$, where ${\tilde{\beta }}_{0}$ is obtained by augmenting ${\tilde{\beta }}_{0,\beta_{0}^{\sup}}$ with zeros at those entries not in $\beta_{0}^{\sup}$.

#### 3.2.3. Iteration via Gradient Descent Scheme

One of the most important steps in solving Equation (10) is to iteratively update the estimated $\tilde{\beta }$ by using a gradient descent scheme, which ultimately searches for the global minimum in each iteration. Based on the Wirtinger derivative [26], the gradient of $f\left(\tilde{\beta }\right)$ in Equation (10) can be defined as the following:

(14) $$\nabla f\left(\tilde{\beta }\right)=\frac{1}{L}\sum_{i=1}^{L}\left({\left|S_{i}\tilde{\beta }\right|}^{2}-Sr_{i}\right)\left(S_{i}{}^{T}S_{i}\right)\tilde{\beta }.$$

Therefore, in the τ-th iteration, the estimated $\tilde{\beta }$ can be updated once in the direction of the negative gradient, which is expressed as Equation (15):

(15) $${\tilde{\beta }}^{\tau +1}={\tilde{\beta }}^{\tau }-\frac{\mu_{\tau }}{\frac{1}{L}\sum_{l=1}^{L}Sr_{l}}\nabla f\left({\tilde{\beta }}^{\tau }\right),$$

where $\mu_{\tau }$ is the stepsize of the τ-th iteration. When τ=0, ${\tilde{\beta }}^{0}$ is obtained from the previous section. In each iteration of Equation (14), a hard threshold operation can be performed on the calculated ${\tilde{\beta }}^{\tau +1}$, which can be written as Equation (16):

(16) $${\tilde{\beta }}^{\tau +1}=T_{k}\left({\tilde{\beta }}^{\tau +1}\right).$$

Here, $T_{k}\left({\tilde{\beta }}^{\tau +1}\right)$ represents an operation that keeps *k* entries of the largest absolute values of ${\tilde{\beta }}^{\tau +1}$ and sets the other entries of ${\tilde{\beta }}^{\tau +1}$ to zero. The hard threshold operation can reduce the freedom dimension and constrain the searching domain [33,34].

#### 3.2.4. Optimizing the Iteration Stepsize

It is worth noting that the iterative update in Equation (15) is performed along the negative gradient direction, and each step of the iteration needs to set a stepsize $\mu_{\tau }$. In References [26,27,28,29,30,31,32,33,34], the stepsize $\mu_{\tau }$ in each iteration is chosen by experience. In the early iterations, a small $\mu_{\tau }$ should be taken due to the large relative error of the estimated $\tilde{\beta }$, but, as the iterations count increases, a larger $\mu_{\tau }$ can be adopted for accelerating the convergence rate. Actually, if $\mu_{\tau }$ is too small, it will cause the algorithm to converge slowly, and if $\mu_{\tau }$ is too large, it will cause the algorithm to diverge. Thus, picking out a reasonable $\mu_{\tau }$ in each iteration is challenging but quite necessary for the algorithm. In order to optimize $\mu_{\tau }$ in each iteration, we can formulate the problem of selecting $\mu_{\tau }$ of the τ-th iteration as the following:

(17) $$\mu_{\tau }=\;\underset{\mu }{\arg\;\min}\;f\left({\tilde{\beta }}^{\tau }-\frac{\mu_{\tau }}{\frac{1}{L}\sum_{l=1}^{L}Sr_{l}}\nabla f\left({\tilde{\beta }}^{\tau }\right)\right).$$

Equation (17) is an optimization problem about the stepsize $\mu_{\tau }$, and many efficient methods can be used to solve it. Here, we adopt a novel method detailed in Reference [35]. By combining Equations (10) and (17), it is easy to get the following equation:

(18) $$\;f\left({\tilde{\beta }}^{\tau }-\frac{\mu_{\tau }}{\frac{1}{L}\sum_{l=1}^{L}Sr_{l}}\nabla f\left({\tilde{\beta }}^{\tau }\right)\right)=\frac{1}{2L}{\sum_{i=1}^{L}\left({\left|S_{i}\left({\tilde{\beta }}^{\tau }-\frac{\mu_{\tau }}{\frac{1}{L}\sum_{l=1}^{L}Sr_{l}}\nabla f\left({\tilde{\beta }}^{\tau }\right)\right)\right|}^{2}-Sr_{i}\right)}^{2}.$$

Obviously, Equation (18) is a univariate quartic. Hence, in order to obtain an optimal $\mu_{\tau }$, the function $f\left({\tilde{\beta }}^{\tau }-\mu_{\tau }\nabla f\left({\tilde{\beta }}^{\tau }\right)/\left(1/L\sum_{l=1}^{L}Sr_{l}\right)\right)$ must satisfy the following optimization condition:

(19) $$\;\frac{df\left({\tilde{\beta }}^{\tau }-\frac{\mu_{\tau }}{\frac{1}{L}\sum_{l=1}^{L}Sr_{l}}\nabla f\left({\tilde{\beta }}^{\tau }\right)\right)}{d\mu_{\tau }}=0.$$

After fully expanding Equation (19), it will be written as Equation (20):

(20) ∑i=1L−ReSiβ˜τ*QS˜riτ−Sri+μτ2ReSiβ˜τ*Q2+Q2S˜riτ−Sri−μτ23ReSiβ˜τ*QQ2+μτ3Q4=0,

where $\tilde{S}r_{i}{}^{\tau }={\left|S_{i}{\tilde{\beta }}^{\tau }\right|}^{2}$, and $Q=S_{i}\nabla f\left({\tilde{\beta }}^{\tau }\right)/\left(1/L\sum_{l=1}^{L}Sr_{l}\right)$. Moreover, ${\left(\cdot \right)}^{*}$ and Re· denote the complex conjugate and the real part of the complex number, respectively. It can be seen from Equation (20) that it is a univariate cubic equation about $\mu_{\tau }$, which can be simply written as the following:

(21) $$h_{4}\mu_{\tau }{}^{3}+h_{3}\mu_{\tau }{}^{2}+h_{2}\mu_{\tau }{}^{1}+h_{1}=0,$$

where the coefficients can be calculated separately by h1=−∑i=1LReSiβ˜τ*QS˜riτ−Sri, h2=∑i=1L2ReSiβ˜τ*Q2+Q2S˜riτ−Sri, h3=−3∑i=1LReSiβ˜τ*QQ2, and $h_{4}=\sum_{i=1}^{L}Q^{4}$.

According to the relevant mathematical knowledge, when the coefficients of the univariate cubic equation are all real values, the form of the root has two possible cases, and we will only adopt its minimum real value [35]. 

Finally, the procedure of the SWFOS algorithm is detailed in Algorithm 1.

**Algorithm 1:** Sparse Wirtinger Flow algorithm with Optimal Stepsize
**Input:**
 Sr: echo signal intensities vector.  S: reference signal matrix.  k: the sparsity of target scattering coefficient vector β.  $\alpha_{y}$: truncation thresholds of initialization.  T: the maximum number of iterations. 
**Support recovery:**
 Set $\beta_{0}^{\sup}$ to include indices corresponding to the k largest instances of ${\left\{\frac{1}{L}\sum_{i=1}^{L}Sr_{i}S_{i,j}^{2}\right\}}_{j=1}^{w}$. 
**Initialization:**
 By 100 power iterations, compute the principal eigenvector ${\tilde{\beta }}_{0,\beta_{0}^{\sup}}$, which corresponds to the  largest eigenvalue of $Y=\frac{1}{L}\sum_{i=1}^{L}Sr_{i}\left(S_{i,\beta_{0}^{\sup}}{}^{T}S_{i,\beta_{0}^{\sup}}\right)1_{\left\{\left|Sr_{i}\right|\leq \alpha_{y}^{2}\left(\frac{1}{L}\sum_{l=1}^{L}Sr_{l}\right)\right\}}$. Set ${\tilde{\beta }}^{0}$ as $\sqrt{1/L\sum_{l=1}^{L}Sr_{l}}{\tilde{\beta }}_{0}$,  where ${\tilde{\beta }}_{0}$ is obtained by augmenting ${\tilde{\beta }}_{0,\beta_{0}^{\sup}}$ with zeros at entries not in $\beta_{0}^{\sup}$. 
**Iteration via a gradient descent scheme:**
 τ=0, ${\tilde{\beta }}^{0}$.  **For**
τ=0 to T−1, do    Set the optimal stepsize $\mu_{\tau }$ by computing the root of the following: 
$$h_{4}\mu_{\tau }{}^{3}+h_{3}\mu_{\tau }{}^{2}+h_{2}\mu_{\tau }{}^{1}+h_{1}=0$$
   where h1=−∑i=1LReSiβ˜τ*QS˜riτ−Sri, h2=∑i=1L2ReSiβ˜τ*Q2+Q2S˜riτ−Sri,    h3=−3∑i=1LReSiβ˜τ*QQ2, and $h_{4}=\sum_{i=1}^{L}Q^{4}$.    Update 
$${\tilde{\beta }}^{\tau +1}={\tilde{\beta }}^{\tau }-\frac{\mu_{\tau }}{\frac{1}{L}\sum_{l=1}^{L}Sr_{l}}\nabla f\left({\tilde{\beta }}^{\tau }\right)$$

$${\tilde{\beta }}^{\tau +1}=T_{\tau }\left({\tilde{\beta }}^{\tau +1}\right)$$
 **End For.**

**Output:**
 ${\tilde{\beta }}^{\tau +1}$: the estimated target-scattering coefficient vector. 

## 4. Numerical Simulation

In this section, the numerical simulation results are presented to illustrate the performance of the SWFOS algorithm and the feasibility of the PR algorithm in PL-TCAI. First, the comparison results between the SWFOS algorithm and other algorithms are given, showing the advantages of SWFOS in some aspects. Second, the reason why the SWFOS algorithm can be adopted in the PL-TCAI system is given, which proves the practicability of SWFOS. Finally, the performance results of the SWFOS algorithm in the PL-TCAI system are given, which demonstrate that the SWFOS algorithm can effectively reduce the number of measurement samples and shorten imaging time at low SNRs. All simulations are performed on a computer with Intel Xeon CPU Gold 5188 at 2.3 GHz and 128 GB of memory. In the simulation, the ratio between the number of samples *L* and the number of grid cells *W* can be written as *L/W*. The imaging performance is evaluated by relative imaging error (RIE) which can be calculated as the following:

(22) $$\mathrm{RIE}=20\log_{10}\left(\frac{{\left\|\tilde{\beta }-\beta \right\|}_{2}}{{\left\|\beta \right\|}_{2}}\right),$$

where $\tilde{\beta }$ is the estimated target scattering coefficient vector and $\left\|\right.\cdot {\left.\right\|}_{2}$ denotes the Euclidean norm of a vector. Here, we adopt the empirical success rate of 100 Monte Carlo trials to evaluate the recovery performance of various algorithms, where a success of a trial is declared if the RIE is below –100 dB. In order to estimate the correlation between different columns of S, we adopt the autocorrelation coefficients of columns, which is also called the space independence function in Reference [9] and is defined as the following:

(23) $$\begin{array}{ccc} \rho_{column}=\frac{\mathrm{cov}\left(S_{\cdot j},S_{\cdot j^{\prime }}\right)}{\sigma \left(S_{\cdot j}\right)\sigma \left(S_{\cdot j^{\prime }}\right)}, & j=1,2,\ldots ,W, & j^{\prime }=\frac{W}{2} \end{array},$$

where $\rho_{column}$ is the autocorrelation coefficients of columns. $S_{\cdot j}$ and $S_{i\cdot }$ are the *j*-th column and the *i*-th row of the matrix S, respectively. Moreover, the operation $\mathrm{cov}\left(A,B\right)$ refers to find the covariance for two vectors A and B, and the operation $\sigma \left(A\right)$ refers to find the standard deviation of vector A.

### 4.1. Performance of SWFOS Algorithm

To demonstrate the performance of the SWFOS algorithm, the SWF, WFOS, SPARTA, TAF, and RAF algorithms are implemented as comparisons. Among them, the sparse PR algorithms include the SWFOS, SWF, and SPARTA algorithms, and the others are non-sparse algorithms. In this simulation, considering only the complex case, we assume that all reference signal vectors ${\left\{S_{i}\right\}}_{1\leq i\leq L}$ satisfy the independent standard complex Gaussian distribution, which can be expressed as ${\left\{S_{i}\right\}}_{1\leq i\leq L}∼N\left(0,I_{W}/2\right)+jN\left(0,I_{W}/2\right)$. We set the target scattering coefficient vector β as a real Gaussian random vector, satisfying $\beta ∼N\left(0,I\right)$ and $\beta \in {\mathbb{R}}^{1000}$. For fairness, the initialization of all algorithms is obtained by 100 power iterations, and a fixed number of gradient iterations *T* = 1000 are run for all algorithms.

**Figure 2:**
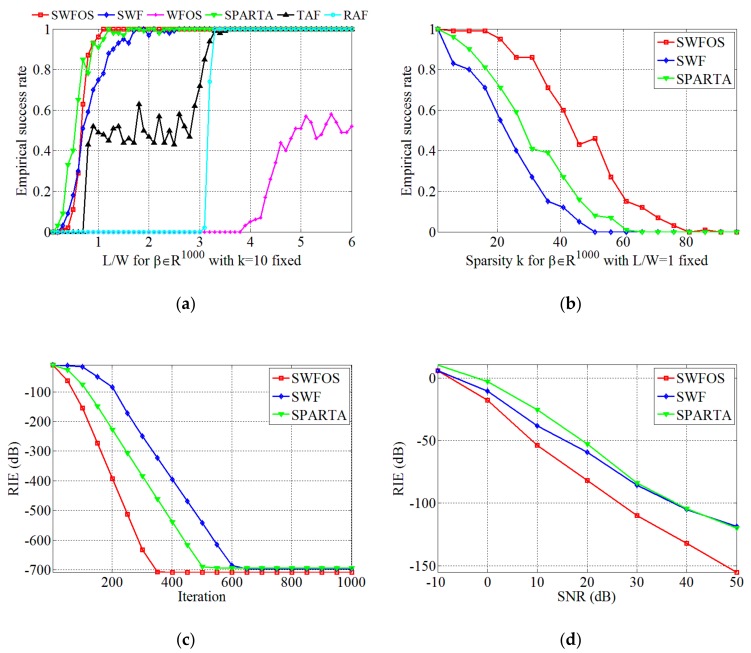
Performance results of the sparse Wirtinger flow algorithm with optimal stepsize (SWFOS) algorithm with other algorithms in the complex case: (**a**) empirical success rates for different *L/W* at fixed *k* = 10; (**b**) empirical success rates for different *k* at fixed *L/W* = 1; (**c**) relative imaging errors (RIEs) for different number of iterations at same condition; and (**d**) RIEs for different SNRs at same condition.

From Figure 2a, we can find that the SWFOS, SWF, and SPARTA algorithms can successfully recover β accurately at a low ratio *L/W* due to the consideration of sparsity priori, while conventional algorithms, such as TAF, RAF, and WFOS algorithms, require a high ratio *L/W*. This intuitively proves that when the target is sparse, the number of samples will be greatly reduced if the target imaging is reconstructed accurately by the sparse algorithm. In addition, we can also carefully observe that the recovery performance of the SWFOS algorithm is slightly better than the SWF and SPARTA algorithms in the complex case. As can be seen from the curves, the SWFOS algorithm has a stable 100% empirical success rate at L/W≥1.1, while the SWF and SPARTA algorithms have a 100% empirical success rate at L/W≥2.6 and L/W≥1.6, respectively. 

Figure 2b is a result of the SWFOS, SWF, and SPARTA algorithms, respectively, recovering β of various sparsity *k*. From Figure 2b, we can see that in the complex case, as the sparsity *k* increases, the empirical success rate of all algorithms decreases significantly. However, we can still find that the SWFOS algorithm is more robust to sparsity *k* than the other two algorithms. The curve visually shows that the empirical success rate of the SWFOS algorithm fluctuates slightly and keeps in the high level when k≤20 and fixed L/W=1. In combination with Figure 2a,b, we can also conclude that the robustness of the SWFOS algorithm to the sparsity *k* can be improved by increasing the ratio L/W.

In order to compare the convergence of these three sparse algorithms, we analyze the RIEs of each iteration of algorithms during operation. As can be seen from Figure 2c, the convergence rate of the SWFOS algorithm, which adopts the optimal stepsize, is significantly faster than that of the SWF and SPARTA algorithms. When k=10 and L/W=1 are fixed in the complex case, the RIE of the SWFOS algorithm decreases to –700 dB after 350 iterations, while that of the SWF algorithm and the SPARTA algorithm falls to the same level after 650 iterations and 500 iterations, respectively. This result demonstrates our analysis in the previous section that the SWFOS algorithm can determine the optimal stepsize and direction in each gradient descent iteration to quickly search for the global minimum.

All of Figure 2a–c is obtained in the absence of noise, while the effects of noise on these sparse algorithms are discussed in Figure 2d. We still fix k=10 and L/W=1, and add Gaussian white noise to the imaging model, which is described as Equation (7). The SNR varies from –10 to 50 dB, and all sparse algorithms perform *T* = 1000 iterations, respectively. From Figure 2d, we can see that all algorithms are sensitive to noise, and the RIEs of all algorithms increase gradually as the SNR decreases. However, even if the SNR is reduced to 10 dB, the three sparse algorithms can still recover β. In addition, it can be seen from the curves that the SWFOS algorithm performs slightly better than the SWF and SPARTA algorithms, especially in high SNR conditions.

### 4.2. Application of SWFOS Algorithm in PL-TCAI

We have mentioned above that the SWFOS algorithm performs well at solving the sparse PR problem when all reference signal vectors ${\left\{S_{i\cdot }\right\}}_{1\leq i\leq L}$ satisfy the independent standard complex Gaussian distribution. However, considering the actual situation, it is difficult for ${\left\{S_{i\cdot }\right\}}_{1\leq i\leq L}$ to meet this condition in the proposed PL-TCAI system. In order to prove the feasibility of the SWFOS algorithm applied to the PL-TCAI system, we analyze the characteristics of the reference signal matrix S derived from the PL-TCAI system. Here, we assume that imaging area in the PL-TCAI system is divided into W=32×32 grid cells, and L=1W samples are obtained by the incoherent detector array, which only receives the intensity information of the echo signal. In the simulations, a THz R-FH signal is used as the transmitting signal, and the basic parameter settings are given in Table 1.

**Figure 3:**
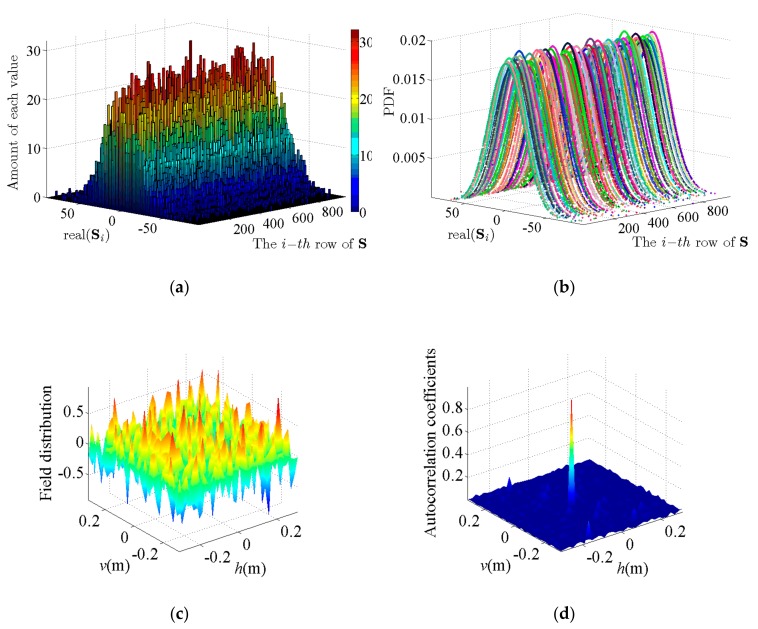
Characteristics of the reference signal that was derived from the PL-TCAI system: (**a**) frequency histogram of the real values of each ${\left\{S_{i\cdot }\right\}}_{1\leq i\leq L}$; (**b**) probability distribution function of the real values of each ${\left\{S_{i\cdot }\right\}}_{1\leq i\leq L}$; (**c**) instantaneous radiation field distribution of PL-TCAI; and (**d**) space independence function of PL-TCAI.

Figure 3a is a three-dimensional frequency histogram, in which can be the data distribution characteristics of each row of S can be visually observed. From Figure 3a, we see that the real values of each row of S is mainly between –50 and 50, and the distribution trend of the amount is gradually decreasing from 0 to both sides. For further analysis, the probability distribution function (PDF) of each row of S is plotted in Figure 3b. As shown by the curves of the different colors in Figure 3b, we can conclude that the real values of each row of S conform to the sub-Gaussian distribution, and the same results also appear in the imaginary values of each row of S. The reason for these results is that we adopt the coded aperture in the PL-TCAI system to randomly modulate the phase of the transmitting signal, making the reference signal corresponding to the imaging area random. According to the central limit theorem (CLT) [40], when directly affected by the random phase modulation, each $S_{i\cdot }$ becomes a random vector and the elements in $S_{i\cdot }$ approximately obey the Gaussian distribution. In fact, the vector $S_{i\cdot }$ is the mathematical expression of the instantaneous radiation field of the reference signal. In order to vividly display the modulated reference signal, we plot the radiation field of the reference signal at a certain moment in Figure 3c. From Figure 3c, we can see that the distribution of the instantaneous radiation field is obviously random, which is consistent with our previous analysis. Furthermore, Figure 3d is the result of the space independence function of the PL-TCAI system, which is calculated from Equation (23). As can be seen from Figure 3d, the columns of S are random and independent, which corresponds to the result that the elements in $S_{i\cdot }$ conform to the random sub-Gaussian distribution. It is worth mentioning that Figure 3c,d can also describe the imaging ability of the PL-TCAI system, where the more random the radiation field is and the better the space independence function is, the stronger the imaging ability is.

**Figure 4:**
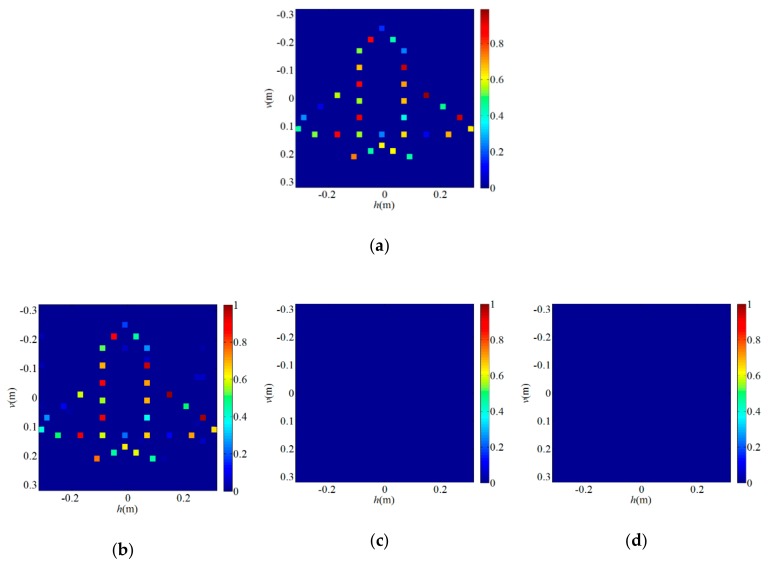
Imaging results for the sparse target when L/W=1.5 in the PL-TCAI system with the setting SNR=20dB: (**a**) original sparse target; (**b**) SWFOS algorithm; (**c**) SWF algorithm; and (**d**) SPARTA algorithm.

**Figure 5:**
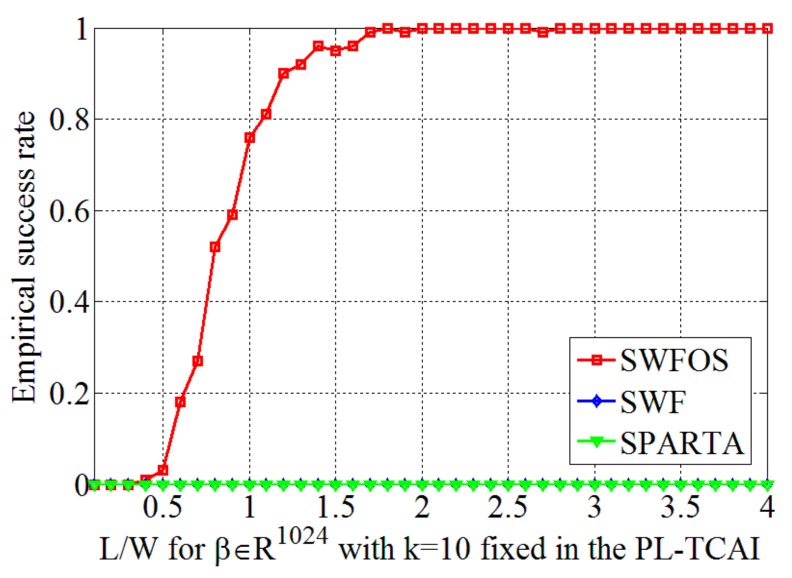
Performance results of the SWFOS algorithm and other sparse algorithms in the PL-TCAI system.

Next, to verify the validity of the proposed algorithm in the PL-TCAI system, we adopt the SWFOS, SWF, and SPARTA algorithms to solve the imaging equation of the PL-TCAI system, where S is derived from the PL-TCAI system but does not conform to the independent standard complex Gaussian distribution. As in Section 4.1, the initialization of all algorithms is obtained by 100 power iterations, and a fixed number of gradient iterations *T* = 1000 are run for all algorithms. Since the priori sparsity *k* of the target is unknown in actual radar imaging, in all sparse algorithms, we set $k\approx \sqrt{W}=32$ as an estimate to ensure a high empirical success rate. *W* is the number of grid cells in the imaging area and is also the dimension of the scattering coefficient vector β. Figure 4a is the original sparse target, and Figure 4b–d shows the imaging results for the sparse target when L/W=1.5 in the PL-TCAI system with the setting SNR = 20 dB. From Figure 4b–d, we can see that only the SWFOS algorithm accurately reconstructs the target, and the other two algorithms do not converge and cannot reconstruct the target. In order to analyze the performance of three sparse algorithms in the PL-TCAI system, we calculate the empirical success rates with L/W increasing. As can be seen from Figure 5, only the SWFOS algorithm can work properly, and the other two algorithms cannot accurately solve the imaging equation. The SWFOS algorithm has a stable 100% empirical success rate at L/W≥1.7, while the SWF and SPARTA algorithms have an 0% empirical success rate for all these ratios. Compared with the ideal S, the performance of the SWFOS algorithm in PL-TCAI is degraded because the vector $S_{i\cdot }$ conforms to the random sub-Gaussian distribution. Because the stepsize of the SWF and SPARTA algorithms is chosen empirically, for an actual PL-TCAI system, when $S_{i\cdot }$ does not satisfy the independent standard complex Gaussian distribution, it is necessary to reselect a new stepsize. However, for the SWF and SPARTA algorithms, it is extremely difficult to select the appropriate stepsize for the PL-TCAI system based solely on experience. Such a dilemma will never appear in the SWFOS algorithm, which can calculate an optimal stepsize in each iteration to accurately solve the imaging equation. 

**Figure 6:**
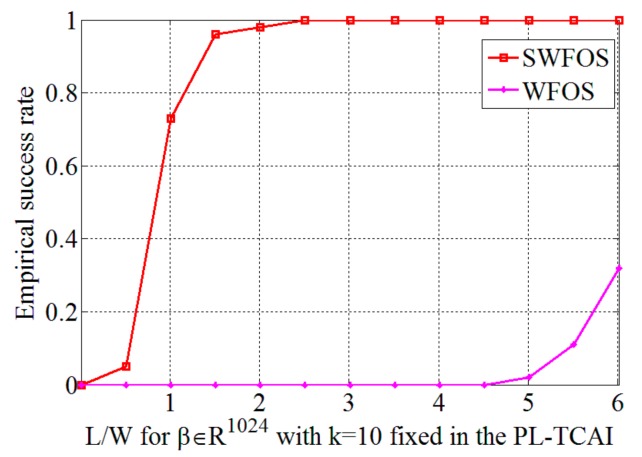
Performance results of the SWFOS algorithm and the WFOS algorithm in the PL-TCAI system.

**Figure 7:**
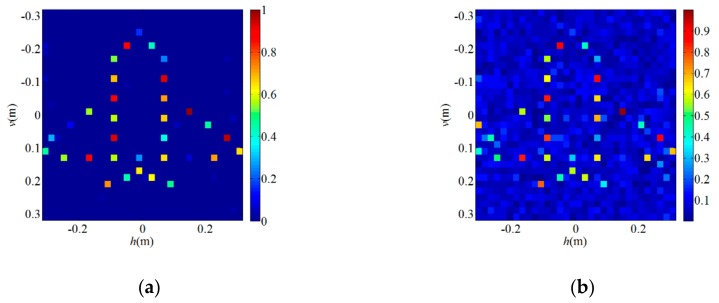
Imaging results for the sparse target in the PL-TCAI system with the setting SNR = 20 dB: (**a**) SWFOS algorithm with L/W=1.5; and (**b**) WFOS algorithm with L/W=6.

Furthermore, in order to verify that the proposed SWFOS algorithm can significantly reduce the number of samples needed for sparse target imaging in the PL-TCAI system, we compare the performance of the SWFOS and WFOS algorithms with different numbers of samples when setting SNR = 20 dB. Figure 6 shows the empirical success rates of SWFOS and WFOS in different L/W ratios. From Figure 6, we can see that, when reconstructing the sparse target, the SWFOS algorithm has a high empirical success rate at L/W=1.5, while, for the WFOS algorithm, without using sparse prior of targets, its empirical success rate is still very low at L/W=6, which is only about 30%. Figure 7 shows the imaging results of the SWFOS algorithm and the WFOS algorithm for the sparse target in the different number of samples, and we can see that the SWFOS algorithm can accurately reconstruct the target even if L/W=1.5. Under the same conditions, the WFOS algorithm will fail to reconstruct the target obviously. Of course, with the increase of L/W, the WFOS algorithm can gradually reconstruct the target. As shown in Figure 7b, most strong scatterers of the target can be reconstructed when L/W=6, but there are still many weak spurious scatterers. To further evaluate the imaging performance of the proposed algorithm in the PL-TCAI system, we record the RIEs and runtime of two algorithms, and the result is shown in Table 2. From Table 2, we can see that, although the SWFOS algorithm which utilizes the sparse prior needs fewer samples to reconstruct the sparse target, it consumes less time and makes the RIE smaller.

**Figure 8:**
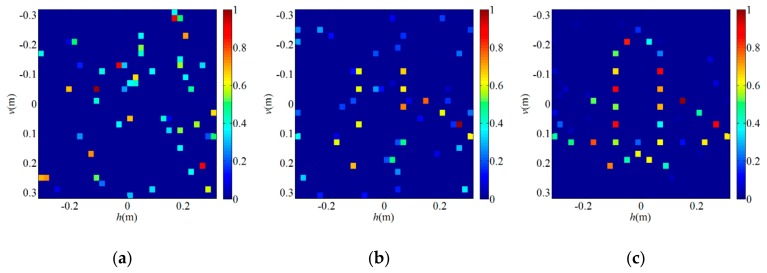
Imaging results of the SWFOS algorithm when L/W=1.5 in the PL-TCAI system with different SNRs: (**a**) SNR = 0 dB; (**b**) SNR = 10 dB; and (**c**) SNR = 15 dB.

**Figure 9:**
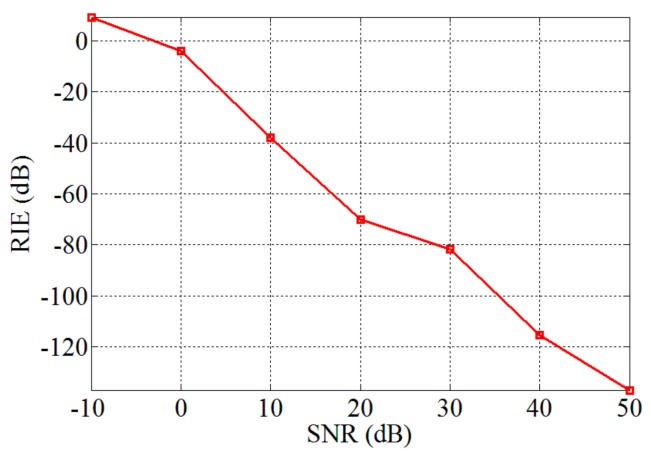
Performance results of the SWFOS algorithm with different SNRs in the PL-TCAI system.

Finally, Figure 8a–c illustrates the imaging results of the SWFOS algorithm for the sparse target when L/W = 1.5 in the PL-TCAI system with different SNRs. From Figure 8, we can find that the sparse target can be fully identified at SNR = 15 dB, while, when SNR = 10 dB, the sparse target can only be partially reconstructed. To analyze the influence of noise on the SWFOS algorithm in the PL-TCAI system, we calculate the RIEs with the SNR increasing when setting L/W=1.5 and k=10, and the result is plotted in Figure 9. From Figure 9, we can see that the SWFOS algorithm can perform well in the PL-TCAI system with low SNRs, such as RIE = –70dB when SNR = 20 dB. With the improvement of SNR condition, the imaging quality of SWFOS algorithm is greatly improved, and it can reconstruct the sparse target accurately. 

## 5. Discussion

Numerical simulation results show that the SWFOS algorithm can solve the Phase Retrieval problem well and can also be effectively applied in PL-TCAI. However, the derivation method of the reference signal $S^{\left(q\right)}(t_{n},r_{w})$ in the PL-TCAI proposed in this paper is based on the distance and time delay, and its accuracy is not completely applicable to the far field. Since the accuracy of reference signal $S^{\left(q\right)}(t_{n},r_{w})$ has a great influence on imaging results of PL-TCAI, ensuring the accuracy of reference signal $S^{\left(q\right)}(t_{n},r_{w})$ will be the main work we are facing in the actual PL-TCAI system. In addition, since the atmospheric water vapor has a high absorption for terahertz waves, it is difficult for PL-TCAI to achieve high-resolution imaging at a long distance in the atmosphere, which is also a huge challenge for all terahertz imaging.

## 6. Conclusions

In this paper, we propose a compact PL-TCAI system configuration based on the combination of the incoherent detector array and the coded aperture, which can achieve fast imaging, high-resolution imaging, and forward-looking imaging without any relative motion. We also propose a sparse phase retrieval algorithm named SWFOS for the sparse target in PL-TCAI, which aims to reduce the number of measurement samples required for imaging. Firstly, the proposed configuration of PL-TCAI is introduced in detail, in which the multiple-output technology adopted by the incoherent detector array can significantly reduce the number of coding and sampling compared with the single detector. Then, according to the proposed system configuration, the imaging equation of PL-TCAI which adopts the R-FH signal as the transmitting signal is derived in detail. Besides, we analyze the target reconstruction principle of PL-TCAI and propose the SWFOS algorithm for sparse target imaging. The specific procedures of the SWFOS algorithm include the support recovery, initialization by truncated spectral method, iteration via gradient descent scheme, hard threshold operation, and iteration stepsize optimization. Finally, numerical simulation results show the basic performance of the SWFOS algorithm and demonstrate the feasibility of applying the SWFOS algorithm to sparse target imaging in PL-TCAI. In conclusion, the proposed structure and algorithm can significantly reduce the amount of coding and sampling required for sparse target imaging, shorten the imaging time of the PL-TCAI system, and provide a new method for high-speed PTCAI, at nearly real-time frame rates.

## Figures and Tables

**Table 1:** Basic parameter settings used in the simulations.

Parameter	Value
Center frequency	345 GHz
Pulse width	100 μs
Bandwidth	10 GHz
Imaging distance	20 m
The size of the coded aperture	0.5 m×0.5 m
The number of elements in the coded aperture	30×30
The number of elements in the incoherent detector array	2×2
The size of the grid cells in the imaging plane	0.02 m×0.02 m
The number of grid cells in the imaging plane	32×32

**Table 2:** Performance comparison of algorithms in PL-TCAI system.

Algorithms	Sparsity *k*	L/W	RIE (dB)	Runtime (s)
SWFOS	33	1.5	−65.294	47.65
WFOS	33	6	−1.0223	174.84

## References

[1] Chen S., Hua X., Wang H., Luo C., Cheng Y., Deng B. (2018). Three-Dimensional Terahertz Coded-Aperture Imaging Based on Geometric Measures. Sensors.

[2] Gollub J.N., Yurduseven O., Trofatter K.P., Arnitz D., Imani M.F., Sleasman T., Boyarsky M., Rose A., Pedross-Engel A., Odabasi H. (2017). Large Metasurface Aperture for Millimeter Wave Computational Imaging at the Human-Scale. Sci. Rep..

[3] Li Y.B., Li L.L., Xu B.B., Wu W., Wu R.Y., Wan X., Cheng Q., Cui T.J. (2016). Transmission-Type 2-Bit Programmable Metasurface for Single-Sensor and Single-Frequency Microwave Imaging. Sci. Rep..

[4] Chen S., Luo C., Wang H., Wang W., Peng L., Zhuang Z. (2018). Three-Dimensional Terahertz Coded-Aperture Imaging Based on Back Projection. Sensors.

[5] Zhou T., Shen F., Xu K., Tang Z., Wang J., Zhang B., Ye D., Huangfu J., Li C., Ran L. (2018). Microwave Imaging Customized on Demand Under Random Field Illumination. IEEE Trans. Microw. Theory Tech..

[6] Watts C.M., Shrekenhamer D., Montoya J., Lipworth G., Hunt J., Sleasman T., Krishna S., Smith D.R., Padilla W.J. (2014). Terahertz compressive imaging with metamaterial spatial light modulators. Nat. Photonics.

[7] Perez-Palomino G., Encinar J.A., Dickie R., Cahill R. Preliminary design of a liquid crystal-based reflectarray antenna for beam-scanning in THz. Proceedings of the 2013 IEEE Antennas and Propagation Society International Symposium.

[8] Perez-Palomino G., Barba M., Encinar J.A., Cahill R., Dickie R., Baine P., Bain M. (2015). Design and Demonstration of an Electronically Scanned Reflectarray Antenna at 100 GHz Using Multiresonant Cells Based on Liquid Crystals. IEEE Trans. Antennas Propag..

[9] Chen S., Luo C., Deng B., Qin Y., Wang H. (2017). Study on coding strategies for radar coded-aperture imaging in terahertz band. J. Electron. Imaging.

[10] Li D., Li X., Qin Y., Cheng Y., Wang H. (2013). Radar Coincidence Imaging: An Instantaneous Imaging Technique with Stochastic Signals. IEEE Trans. Geosci. Remote. Sens..

[11] Arnone D., Ciesla C., Pepper M. (2000). Terahertz imaging comes into view. Phys. World.

[12] Luo C.-G., Deng B., Wang H.-Q., Qin Y.-L. (2019). High-resolution terahertz coded-aperture imaging for near-field three-dimensional target. Appl. Opt..

[13] Chen S., Luo C., Deng B., Wang H., Cheng Y., Zhuang Z. (2018). Three-Dimensional Terahertz Coded-Aperture Imaging Based on Single Input Multiple Output Technology. Sensors.

[14] Cooper K.B., Dengler R.J., Llombart N., Thomas B., Chattopadhyay G., Siegel P.H. (2011). THz Imaging Radar for Standoff Personnel Screening. IEEE Trans. Terahertz Sci. Technol..

[15] Dickmann J., Klappstein J., Hahn M., Appenrodt N., Bloecher H., Werber K., Sailer A. Automotive radar the key technology for autonomous driving: From detection and ranging to environmental understanding. Proceedings of the 2016 IEEE Radar Conference.

[16] Rogalski A., Sizov F. (2011). Terahertz detectors and focal plane arrays. Opto-Electron. Rev..

[17] Shan W., Yang J., Shi S., Yao Q., Zuo Y., Lin Z., Chen S., Zhang X., Duan W., Cao A. (2012). Development of Superconducting Spectroscopic Array Receiver: A Multibeam 2SB SIS Receiver for Millimeter-Wave Radio Astronomy. IEEE Trans. Terahertz Sci. Technol..

[18] Kawamura J., Tong C.-Y.E., Blundell R., Papa D.C., Hunter T.R., Patt F., Gol’Tsman G., Gershenzon E. (2001). Terahertz-frequency waveguide NbN hot-electron bolometer mixer. IEEE Trans. Appl. Supercond..

[19] Yurduseven O., Fromenteze T., Smith D.R. (2018). Relaxation of Alignment Errors and Phase Calibration in Computational Frequency-Diverse Imaging using Phase Retrieval. IEEE Access.

[20] Peng L., Luo C., Deng B., Wang H., Qin Y., Chen S. (2019). Phaseless Terahertz Coded-Aperture Imaging Based on Incoherent Detection. Sensors.

[21] Miao J., Charalambous P., Kirz J., Sayre D. (1999). Extending the methodology of X-ray crystallography to allow imaging of micrometre-sized non-crystalline specimens. Nature.

[22] Fienup C., Dainty J. (1987). Phase retrieval and image reconstruction for astronomy. Image Recovery Theory Appl..

[23] Chai A., Moscoso M., Papanicolaou G. (2010). Array imaging using intensity-only measurements. Inverse Probl..

[24] Gerchberg R.W. (1972). A practical algorithm for the determination of phase from image and diffraction plane pictures. Optik.

[25] Candes E.J., Strohmer T., Voroninski V. (2013). Phaselift: Exact and stable signal recovery from magnitude measurements via convex programming. Commun. Pure Appl. Math..

[26] Candés E.J., Li X., Soltanolkotabi M. (2015). Phase Retrieval via Wirtinger Flow: Theory and Algorithms. IEEE Trans. Inf. Theory.

[27] Chen Y., Candès E.J. (2017). Solving random quadratic systems of equations is nearly as easy as solving linear systems. Commun. Pure Appl. Math..

[28] Kolte R., Özgür A. (2016). Phase retrieval via incremental truncated Wirtinger flow. arXiv.

[29] Zhang H., Zhou Y., Liang Y., Chi Y. (2017). A nonconvex approach for phase retrieval: Reshaped wirtinger flow and incremental algorithms. J. Mach. Learn. Res..

[30] Wang G., Giannakis G.B., Eldar Y.C. (2017). Solving systems of random quadratic equations via truncated amplitude flow. IEEE T. Inform. Theory.

[31] Wang G., Giannakis G.B., Saad Y., Chen J. (2018). Phase Retrieval via Reweighted Amplitude Flow. IEEE Trans. Signal Process..

[32] Cai T.T., Ma Z., Li X. (2016). Optimal rates of convergence for noisy sparse phase retrieval via thresholded Wirtinger flow. Ann. Stat..

[33] Wang G., Giannakis G.B., Chen J., Akçakaya M. SPARTA: Sparse phase retrieval via truncated amplitude flow. Proceedings of the 2017 IEEE International Conference on Acoustics, Speech and Signal Processing.

[34] Yuan Z., Wang H., Wang Q. (2019). Phase retrieval via Sparse Wirtinger Flow. J. Comput. Appl. Math..

[35] Rajan S., Liu X., Jiang X. (2016). Wirtinger Flow Method with Optimal Stepsize for Phase Retrieval. IEEE Signal Process. Lett..

[36] Headland D., Niu T., Carrasco E., Abbott D., Sriram S., Bhaskaran M., Fumeaux C., Withayachumnankul W. (2016). Terahertz reflectarrays and nonuniform metasurfaces. IEEE J. Sel. Top. Quantum Electron..

[37] Cui T.J., Qi M.Q., Wan X., Zhao J., Cheng Q. (2014). Coding metamaterials, digital metamaterials and programmable metamaterials. Light. Sci. Appl..

[38] Chen S., Luo C., Deng B., Qin Y., Wang H., Zhuang Z. (2018). Research on Resolution of Terahertz Coded-aperture Imaging. J. Radars.

[39] Huang J. Bandwidth study of microstrip reflectarray and a novel phased reflectarray concept. Proceedings of the IEEE Antennas and Propagation Society International Symposium.

[40] Hoeffding W., Robbins H. (1948). The central limit theorem for dependent random variables. Duke Math. J..

